# Mitogenic and functional responses by nicotine and hydrogen peroxide in AR42J cells: a comparative study

**DOI:** 10.1186/1617-9625-4-5

**Published:** 2008-07-31

**Authors:** Azida Walker, Kodetthoor B Udupa, Parimal Chowdhury

**Affiliations:** 1 Department of Physiology and Biophysics, University of Arkansas for Medical Sciences, Little Rock, AR, USA; 2 Department of Geriatrics, University of Arkansas for Medical Sciences, Little Rock, AR, USA; 3 Medical Research, Central Arkansas Veterans Healthcare System, Little Rock, AR, USA

## Abstract

The aim of the current study was to investigate the oxidative effects of nicotine by examining the mitogenic and functional responses in AR42J cells. As a control and for comparison, hydrogen peroxide (H_2_O_2_) was used as a source of known oxidative biomarker. Responses were examined by determining cell proliferation through the activation of ERK signaling, basal and CCK-stimulated cell function and measuring lipid peroxidation. AR42J cells have been exposed to either a non-cytotoxic dose of 20 μM H_2_O_2 _for 15 min or to 100 μM of nicotine for 3 min respectively. Nicotine and H_2_O_2 _at these dose and time intervals produced similar levels of malondialdyde (MDA) production and p-ERK1/2 activation. Immunofluorescence studies employing specific antibody to p-ERK1/2 confirmed the latter. Nicotine-induced increase in the proliferation of AR42J cells was significantly higher in comparison to H_2_O_2 _exposed cells. CCK-stimulated cell function induced by nicotine was significantly higher in AR42J cells as compared to the response by H_2_O_2_. These results suggest that nicotine- induced mitogenic and functional response in AR42J cells are associated with ERK signaling and increase in reactive oxygen species production. The data suggests that nicotine-induced mitogenic response in AR42J cells closely identifies the response induced by an oxidative biomarker.

## Background

Nicotine, one of the main chemicals in tobacco, has been known as a primary psychoactive ingredient that is responsible for the reinforced behavior in smokers. Each year in the United States, 435,000, or 1 in every 5 deaths, are attributed to cigarette smoking [1]. About half of the young adult smokers today who continue to smoke throughout their life will die of a smoke related diseases [2]. Further, it has been shown that smoking is an independent risk factor in the development of chronic pancreatitis and pancreatic cancer [3,4]. In animal studies it has been shown that nicotine plays a role in the induction of pathophysiology of pancreas [5,6].

Evidence shows that lipid peroxidation occurs in pancreatic tissues when exposed to nicotine [7] and that the mitochondrial respiratory chain is affected by nicotine leading to an increased generation of superoxide anions and hydrogen peroxide [8]. Clinical studies have indicated that patients with acute pancreatitis have a higher plasma levels of lipid peroxide than that observed in patients with mild pancreatitis [9]. This suggests that multiple etiological factors other than the release of enzymes may be responsible in this mechanism. As of to-date, however, there have been no reported studies investigating the role of oxyradicals induced by nicotine in the pancreas, and to determine whether oxyradical formation by nicotine contribute to the pathophysiological mechanisms associated with pancreatic injury encountered in smokers We have shown earlier that nicotine induces functional alterations and MAP kinase signaling pathways in pancreatic acinar cells [10,11]; however, the underlying mechanisms responsible for these observed effects by nicotine are still not completely understood. We surmise in this study that nicotine induces the oxidative stress in pancreatic acinar cells and thus contributes to this mechanism.

Oxidative stress arises when there is an imbalance between the formation of reactive oxygen species (ROS) and removal of oxyradicals by scavenging antioxidants. Increase in ROS production has been directly linked to the oxidation of cellular macromolecules, which may cause direct cellular injury or induce a variety of cellular responses through the generation of secondary metabolic reactive species [12]. Clinical studies have shown that oxidative stress leading to lipid peroxidation appears to be linked to the pathogenesis of chronic pancreatitis [13]. Other evidence showing the production of large amounts of oxygen radicals in lymphocytes due to cigarette smoking [14] suggests that nicotine derived from cigarette smoking may play a role, in pathophysiological process.

The current study was designed to examine whether exposure of AR42J cells to nicotine causes the production of reactive oxygen species. Thus we have reexamined the mitogenic and functional responses of this cell line to nicotine and compared its effects with hydrogen peroxide (H_2_O_2_), a known oxidative biomarker, in the same cellular system in order to evaluate the direct effect of oxidative stress in this cell line. The AR42J cell line was used because of its stability as an immortal tumor cell line and its known similarity in physiological characteristics to primary acinar cells [15].

## Materials and methods

### Cell Culture

AR42J cells, a rat pancreatic tumor cell line, were obtained from ATCC (Rockville, MD). These cells were grown in 75 cm^2 ^flasks with 12 ml of Ham's F12 nutrient media with 2 mM L-glutamine and 1.5% NaHCO_3 _(F12K, obtained from Hyclone, Logan, UT), to which 10% fetal bovine serum (FBS) and 1% penicillin-streptomycin were added. The flasks were kept in an incubator maintained at 37°C with a 5% CO_2_/95% air atmosphere until they reached over 80% confluency.

### Assay of Cell Cytotoxicity in the presence of H_2_O_2_

The cytotoxic effect of H_2_O_2 _on the cells was measured using an LDH-Cytotoxicity Assay Kit from Biovision Research Products (Mountain View, CA). The kit consisted of a lyophilized catalyst and a dye reagent. The test samples were prepared with various doses of H_2_O_2 _ranging from 10–100 μM and cell cytotoxicity measurements were performed following the recommendation of the manufacturer. The cytotoxicity study with nicotine varying doses has been reported previously in this cell system [10]. A dose of 100 μM nicotine was found to be non-toxic. The percent of cytotoxic cells increased significantly beyond this dose level of nicotine. Thus this dose of nicotine was selected for this comparative study.

### Measurement of cellular lipid peroxidation products induced by H_2_O_2 _and nicotine

Lipid peroxidation assay was conducted using MDA-586 method (Oxis Research, Portland, OR) with whole cell lysates obtained after treatment with nicotine or H_2_O_2_. Malondialdehyde (MDA, Sigma-Aldrich, St Louis, MO) was used as a standard. Both the standards and whole cell lysates were incubated for 1 h in a 45°C water bath with N-methyl-2-phenylindole (NM2P, dissolved in acetonitrile) and diluted with methanol together with concentrated HCl. The ratio of cell lysate to the volume of NM2P solution was 1:5. Methods of measurement of MDA from tissue homogenates and blood have been reported recently from our laboratory [16,17].

### MAPK Signaling assay by Western Blot Analysis

Whole cells lysates were prepared from flasks containing more than 80% confluent cells that were tyrpsinized. About 1–2 × 10^6 ^cells were plated per flask. The cells were allowed to attach. The cells were incubated overnight in serum free media. Cells were treated with 100 μM nicotine or 20 μM H_2_O_2_, washed with cold PBS and placed on ice. Two hundred fifty microliters of RIPA buffer containing PMSF/protease III cocktail inhibitor was added. The cells were lysed, sonicated and incubated on ice for 40 min. The cell protein mixture was then spun down at 12,000 rpm for 10 min; supernatant removed and kept on ice. Protein concentration was determined by Bradford assay with bovine serum-albumin as the standard [18].

For Western Blot analysis, a total of 40 μg of cellular protein was loaded onto 12% SDS-polyacrylamide gels and electrophoresed for 1/12 h at a steady voltage of 120 V. The separated protein bands were then transferred to nitrocellulose membranes (BioRad Laboratories, Hercules, CA). The primary antibodies used for probing the nitrocellulose membrane overnight were obtained from Cell Signaling (Danvers, MA). The antibodies used were: anti-ERK1/2, anti-pERK1/2. Subsequently membranes were probed with horseradish peroxidase-conjugated secondary antibody (Pierce Biotechnology Inc, Rockford, IL). Enhanced chemiluminescence (ECL+, Amersham BioSciences, Piscatway, NJ) was used to visualize the bands. The band intensity was quantified using a STORM 860 Imager (Molecular Dynamics, Inc, Sunnyvale CA).

### Cell Proliferation Assay

Cell proliferation studies were conducted after treatments with 100 μM nicotine, or 20 μM H_2_O_2 _using commercially available Cell Viability and Cytotoxicity Assay Kit (Cell Counting Kit, CCK-8, Dojindo Molecular Technologies Inc. Gaithersburg, MD). Ninety six-well microplates were used and 2 × 10^4 ^cells per well were plated. The cells were allowed to attach for 24 h in media containing 10% FBS. Following this, the cells were kept in 0.05% FBS containing media overnight before being treated with 100 μM nicotine or 20 μM H_2_O_2_. Twenty μl of CCK-8 dye was added to each well at specified time interval and incubated further for 3 h at 37°C. The absorbance was measured at 450 nm.

### Localization of MAPK signals measured by Immunofluorescence Imaging

For these studies, 4 × 10^4 ^cells per well were plated in 4-well Lab-Tek chamber slides (Becton Dickinson Labware, Franklin Lakes, NJ). The cells were allowed to attach for 24 h in 10% FBS media before transferring to serum free media overnight. The cells were then exposed to 100 μM nicotine for 3 min or 20 μM H_2_O_2 _for 15 min. After washing briefly with cold PBS, the cells were fixed with 2% paraformaldehyde for 20 min at room temperature, permeablized with 1% Triton X-100 in PBS for 5 min followed by extensive washing with PBS. Blocking was done using 1% bovine serum-albumin and 5% goat serum in PBS. Incubation with primary antibody to p-ERK (1:100 dil) in 1% bovine serum-albumin was continued for 24 h at 4°C. Following incubation the slides were washed 3 times, 10 min each with PBS. After washing, the cells were incubated with fluorescein isothiocyanate-conjugated anti-rabbit IgG antibody (1:50 dilution, Sigma, St. Louis MO), at room temperature for 45 min. Slides were then washed extensively (3 times for 10 min) in PBS. Mounting media from Invitrogen Technologies (Carlsbad, CA) was used to mount the samples. The slides were then viewed under confocal microscope and images were analyzed using Fluorescan 2 software (Fluorescan Labsystems OY, Helsinki, Finland). The negative controls for immunostaining were cells that were unexposed and incubated with secondary antibody alone.

### Assay of basal and stimulated cell function by bioassay

For cell function studies, the cells were grown to 80% and above in confluency. The flasks containing an average 4–6 × 10^6 ^cells, were washed with Hepes-Ringer Buffer (HRB) before treating with 100 μM nicotine in HRB for 3 mins or 20 μM H_2_O_2 _in HRB for 15 min. After incubation the cells were washed with HRB. The cells were then trypsinized using 1 × Trypsin EDTA (Mediatech Inc, Herndon, VA). 5 mls of HRB were then added and the cells were centrifuged for 5 min at 1000 rpm. The supernatant was discarded and the cells were resuspended in HRB. The resuspended cells were incubated with and without CCK (10^-10 ^M) for 30 min at 37°C. After the incubation, the media was removed following centrifugation. Amylase activity was measured employing procion yellow starch as substrate (PRO Chemical & Dye; Somerset, MA) using the method of Jung [19]. The cell pellets were washed with ice cold PBS, lysed with water by sonication and centrifuged. The cell lysate was analyzed for both amylase and protein content.

### Statistical Analysis

Experimental values are calculated as mean ± SEM of the number of experiments indicated in the legends. Data were evaluated for statistical significance with one-way ANOVA. A p-value of 0.05 or less was considered as statistically significant.

## Results

### Effects of H_2_O_2 _on Cell Toxicity

**Figure 1:**
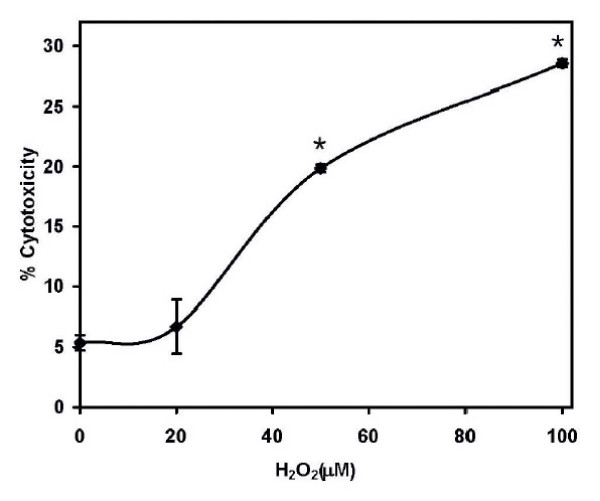
**Measurement of cytotoxic effect of H_2_**O_**2**_. The cells were exposed to H_2_O_2 _in the dose range of 10–100 μM. A BioVision LDH-Cytotoxiciy Assay Kit (Mountain View, CA) was used, and the absorbance was measured after 24 hr incubation in 96-well plates at a wavelength of 495 nm. The percentage cytotoxicity was calculated as the ratio of absorptions of wells treated with H_2_O_2 _and untreated wells; N = 8, *, P < 0.05, significantly different from the uncxposed control.

In order to determine the cytotoxic dose of H_2_O_2 _on AR42J cells, cells were exposed for 24 h to a graded dose of H_2_O_2_. The percent cytotoxicity as determined by LDH release is shown in Figure 1. In untreated control cells the percent of LDH release was 5.4 ± 1.1% while cells exposed to 20 μM H_2_O_2 _it was 6.7 ± 3.9%. This value was not significantly different from the control. As the concentration of H_2_O_2 _increased beyond 20 μM, the percentage of cytotoxic cells was increased. With 100 μM H_2_O_2_, it was 28.0 ± 0.3%. This is not surprising since it has been reported that EL-4 murine lymphoma cells exposed to H_2_O_2 _doses beyond 20 μM, increased number of cytotoxic cells was found by Zhou et al [20]. Thus we have used this non-cytotoxic concentration of 20 μM of H_2_O_2 _in all the subsequent studies.

### Induction of lipid peroxidation by H_2_O_2 _and nicotine in AR42J cells

**Figure 2:**
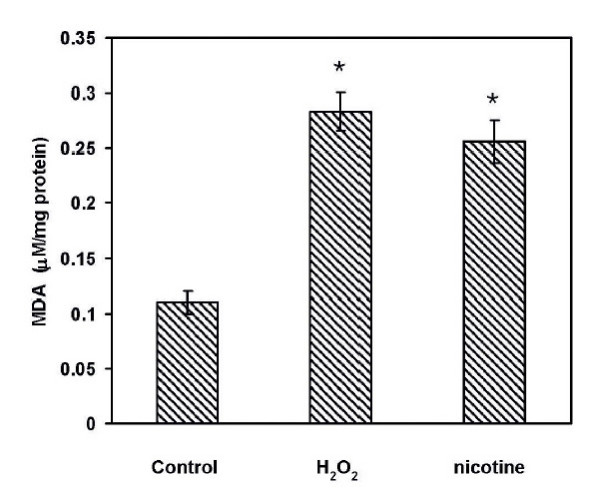
**Lipid peroxidation represented by MDA concentration in AR42J cells**. Control untreated cells, cells exposed to 20 μM H_2_O_2 _for 15 min or to 100 μM nicotine for 3 min were lysed after treatments and the lysates were used in an MDA-586 Assay (Oxis Research, Portland, OR). The values are expressed as μM/mg protein in y-axis. The vertical bar represents treatments with H_2_O_2 _or nicotine, N = 5, *, P < 0.05, significantly different from the uncxposed control.

To determine whether H_2_O_2 _and nicotine would affect the cell's ability to accumulate reactive oxygen species as measured by the production of malondialaldehyde (MDA) within the cells, cells were treated with either H_2_O_2 _(20 μM) for 15 min or with nicotine (100 μM) for 3 min, a non cytotoxic dose determined earlier [10]. Cell lysates were used for the measurement of MDA production as described in the Materials and Methods section. As shown in Figure 2, the concentration of MDA measured in cells exposed to H_2_O_2 _or nicotine were 0.28 ± 0.02 μmol/mg and 0.26 ± 0.02 μmol/mg, respectively. These values were significantly higher than the value of 0.11 ± 0.01 μmol/mg in control cells (p < 0.01).

### Activation of ERK signaling by H_2_O_2_

**Figure 3:**
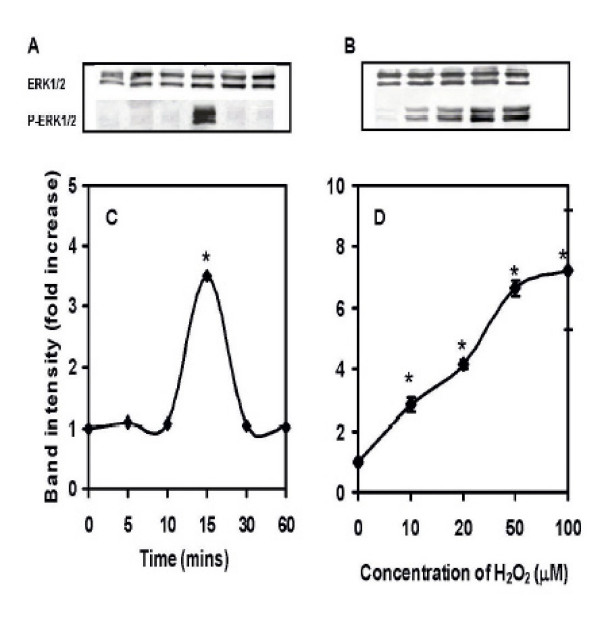
**Dose and time dependent induction of ERK1/2 in AR42J cell**s. Cell lysates were loaded onto an SDS gel, separated by electrophoresis, blocked in 1% fat free milk and probed with antibodies to total and phosphorylated (p) ERK1/2. Horseradish peroxidase-coupled anti IgG was used as a secondary antibody. Bands were visualized with ECL-plus and quantified using a STORM Imager. The data shown as means ± SEM of n = 5 experiments. **A: **Induction of ERK1/2 in cells exposed to 20 μM H_2_O_2 _for 10–60 min compared to control untreated cells. **B: **Induction of ERK in cells exposed to 10–20 μM H_2_O_2 _for 15 min. **C: **Band intensity showing the fold increase in the time dependent induction of ERK1/2. **D: **Band intensity showing the fold increase in the dose dependent induction of ERK1/2.

It has been shown that in other cells, ERK1/2 is activated by H_2_O_2 _treatment [20,21]. To determine the effects of H_2_O_2 _on ERK1/2 activation in AR42J cells, the cells were exposed to H_2_O_2 _(20 μM) for various times. Cell lysates were prepared and measured for total and p-ERK1/2 activation employing the specific antibodies to total and p-ERK1/2 and analyzed by Western blots. With 20 μM H_2_O_2_, a 3 fold increase in band intensity was observed at 15 min which was significantly higher (p < 0.02) when compared to the control cells (Figure 3A). Expressing band intensity as the fold increase above the control, a steady increase in p-ERK activation was observed with increasing concentrations of H_2_O_2 _at 15 min of incubation, attaining a 7-fold increase with 100 μM H_2_O_2 _(Fig. 3B). Total ERK1/2 in all instances showed no alteration with H_2_O_2 _incubation and indicated a uniformity of loading of the wells with samples.

### Response of H_2_O_2 _and nicotine in activation of ERK signaling in AR42J cells

**Figure 4:**
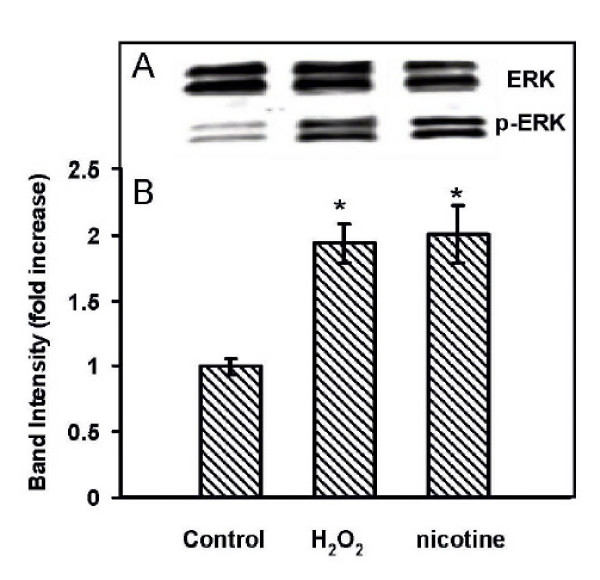
**Induction of ERK1/2 in AR42J cells exposed to nicotine or H_2_O_2_.** Control untreated cells, cells exposed to 20 μM H_2_O_2 _for 15 min or 100 μM nicotine 3 min were lysed and the lysates were used for Western blotting. **A: **Western blot visualization with ECL-plus using STORM Imaging software. **B: **Band intensity showing the fold increase as mean ± SEM of n = 5 experiments. *, P < 0.05, significantly different from the uncxposed control.

The non cytotoxic dose of hydrogen peroxide as determined from the study described above was used in this study along with the non-cytotoxic dose of nicotine reported earlier from this laboratory [10]. The concentration of nicotine used in this study was 100 μM with an incubation time of 3 min. A comparison of p-ERK1/2 activation of cells exposed to nicotine or H_2_O_2 _is shown in Figure 4. The data show that there were increases in p-ERK1/2 activation in cells exposed to 100 μM nicotine for 3 min or 20 μM H_2_O_2 _for 15 min. The band intensities expressed as the fold increase were 2 fold higher with both nicotine and H_2_O_2 _and were significantly higher when compared to the unexposed cells (p < 0.05).

### Cytoplasmic localization of activated p-ERK1/2 by H_2_O_2 _and nicotine as determined by immunofluorescence

**Figure 5:**
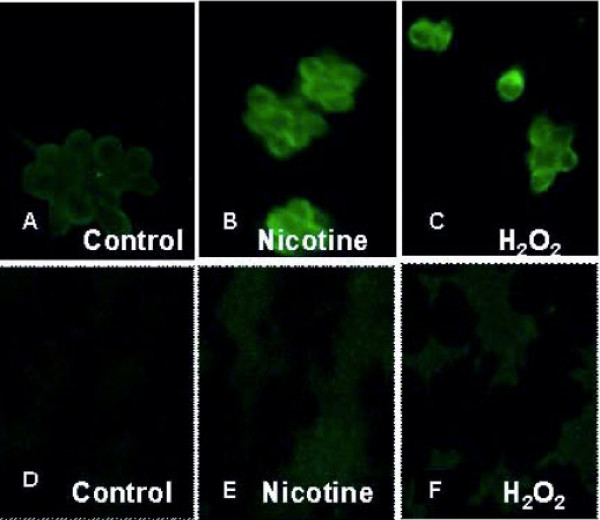
**Induction of p-ERK1/2 in AR42J cells as indicated by immunohistochemistry**. AR42J cells were grown and treated with 100 μM nicotine for 3 min; 20 μM H_2_O_2 _for 15 min. The cells were fixed with paraformaldehyde and treated with antibody to p-ERK1/2 for 1 h. After washing, the cells were treated with secondary antibody labeled FITC. Slides were observed using a confocal microscope **A: **Control untreated cells probed with primary antibody to p-ERK1/2 **B: **Cells exposed to nicotine for 3 min probed with primary antibody to p-ERK1/2 **C: **Cells exposed to H_2_O_2 _for 15 min probed with primary antibody to p-ERK1/2. Negative controls were done by incubating in FITC labeled secondary antibody only. (D – F). **D: **Control untreated cells. **E: **Cells exposed to nicotine for 3 min. **F: **Cells exposed to H_2_O_2 _for 15 min. The experiment was repeated in a set of four under each treatment.

Activation of pERK1/2 in AR42J cells by nicotine and H_2_O_2 _were onfirmed by immunofluorescence study after exposing the cells to nicotine or H_2_O_2_, and probing the fixed cells with antibody to p-ERK1/2. As shown in Figure 5, immunostaining revealed that the p-ERK1/2 signals were distributed throughout the cell cytoplasm with considerably higher fluorescent intensities of p-ERK1/2 observed when cells were treated with nicotine or H_2_O_2_. Cells not exposed to nicotine or H_2_O_2_, on the other hand, showed less fluorescent intensities compared with treated cells. The immunofluorescence data supported the observation of increased induction of pERK1/2 by nicotine and H_2_O_2 _as analyzed by Western Blot.

### Effects of H_2_O_2 _or nicotine on AR42J cell Proliferation

**Figure 6:**
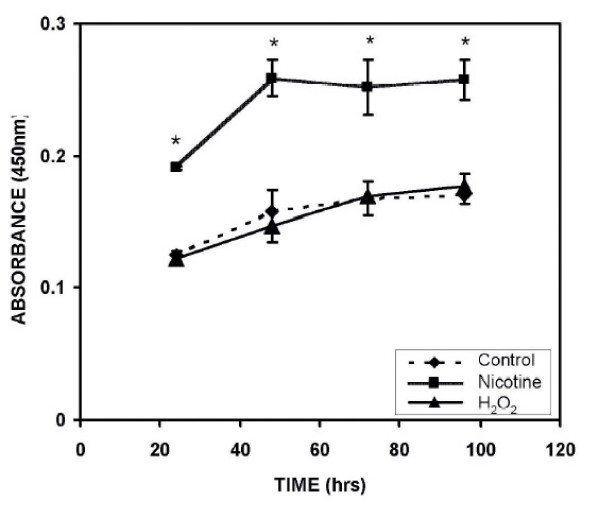
**The effect of nicotine or hydrogen peroxide on the proliferation of AR42J cells**. The cells were plated in 96-well plates and allowed to attach overnight before transferring to 0.05% serum media for 10–12 h before beginning of the study. The cells were then treated with 100 μM nicotine or 20 μM H_2_O_2 _and the proliferation measured at 24–96 h using a cell counting kit from Dojindo Molecular Technologies according to manufacturer's instructions. The data points represent mean ± SEM of 5 experiments. ◆,-- control unexposed cells; ■, --- Nicotine treated; ▲ --, H_2_O_2 _treated. *, P < 0.05, significantly different from the uncxposed control.

In order to compare the nicotine-induced ERK1/2 activation in the growth of AR42J cells with that of H_2_O_2_-induced ERK1/2, cell proliferation experiments were performed. The details of the experiments were described in the Materials and Methods section. The cell proliferation was evaluated from 24–96 h of incubation in media containing 0.05% serum and using an MTT assay kit. The absorbance was measured at 450 nm. As shown in Figure 6, there was a significant difference in proliferation pattern between nicotine exposed cells; the cells treated with H_2_O_2 _or unexposed control cells. The maximum proliferation occurred at 48 h interval and then declined at later periods. The absorbance for nicotine exposed cells at 48 h interval was 0.3 ± 0 arbitrary units while that for controls it was 0.2 ± 0.0 arbitrary units (p < 0.05). There was no significant difference between the proliferation observed for cells exposed to H_2_O_2 _and the control cells.

### Influence of H_2_O_2 _and nicotine Cell Function

**Figure 7:**
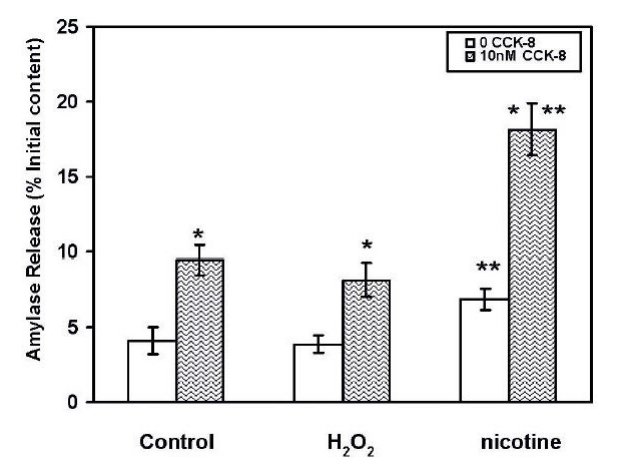
Comparison of cell function of AR42J cells when exposed to nicotine or H_2_O_2_. The cells were treated with 100 μM nicotine for 3 min or 20 μM H_2_O_2 _15 min. Cells were subsequently washed with Hepes-Ringer and incubated at 37°C with or without CCK-8 (10 nM) for 30 min. Amylase released into the incubation medium was measured with procion yellow starch as a substrate. The data are expressed as percent initial content and represent mean ± SEM of 5 experiments. open bar---control unexposed; hatched bar---- CCK-8 stimulated; *, p < 0.05, significantly different from the control; **, p < 0.05, significantly different from H_2_O_2 _exposed cells.

The effects H_2_O_2 _and nicotine on cell function were assessed by determining their ability to stimulate the secretagogue induced enzyme release. The concentrations of nicotine and H_2_O_2 _used in the studies were 100 μM and 20 μM for a time of exposure of 3 and 15 min respectively. The washed cells were incubated with or without cholecystokinin (CCK) at their maximal stimulating concentration of 10^-10 ^M as reported earlier [22]. The release of amylase in response to CCK was measured as percent of initial content and is shown in Figure 7. The percent of amylase released from the cells treated with nicotine was (18.2 ± 1.8% of initial content) and it was significantly higher from the percent amylase released from either the control cells (9.5 ± 1.0.% of initial content, or from the H_2_O_2 _treated cells (8.2 ± 1.1% of initial content, p < 0.05).

## Discussion

The effects of nicotine on cell proliferation and secretion has been reported in this cell line [10]. The current study re-examined the effects of nicotine in the same cell system with the added exposure to hydrogen peroxide to determine whether nicotine-induced effects on these cells are regulated via oxidative stress pathway. Thus hydrogen peroxide was selected as a well known marker for oxidative damage. Previous studies have shown that exposure of human osteosarcoma cell line to 1–10 mM H_2_O_2 _induced reactive oxygen species (ROS) formation, DNA damage, dysfunction of the mitochondrial membrane potential, and early apoptotic changes in this cell line [23]. Since the presence of excessive ROS is known to cause cellular damage by hydroxyl radical attack [24] it was imperative to perform dose response studies with H_2_O_2_.

Cell cytotoxicity experiments have been first performed to determine the effects of H_2_O_2 _at dose-range from 10 – 100 μM. The results show that for concentrations beyond 20 μM, the percentage cytotoxicity was significantly higher than the control and as high as greater than 20%. Other studies have also confirmed an increase in cell toxicity beyond 20 μM [25]. Thus, in order to avoid any cell injury and to maintain the cells within the physiological range, this concentration of H_2_O_2 _was selected for the current study.

Lipid peroxidation occurs through free radical attacks of poly-unsaturated fatty acids leading to formation of lipid hydroperoxides as well as conjugated dienes and aldehydes such as malondialdehyde (MDA). In order to investigate whether nicotine induces the generation of oxygen free radicals within the cell, the lipid peroxidation was measured in response to nicotine and H_2_O_2. _The data from our experiments showed that both nicotine and H_2_O_2 _had significant increases in the concentrations of MDA formation as compared to the control untreated cells suggesting that both nicotine and H_2_O_2 _induced ROS formation in AR42J cells. It has been shown that ROS formation above a critical level in oligodendrocytes is followed by an increase in anti-oxidant enzymes possibly to scavenge oxidative by products such as H_2_O_2 _[26-28]. This mechanism is important for cell survival [28]. In this study we show that the concentration of hydrogen peroxide that induced MDA formation also induced the activation of p-ERK1/2 signaling (Figure 3). These results are consistent with the data on p-ERK1/2 activation as reported in cultured endothelial cells in which peak responses of p-ERK activation is shown to occur after 15 min of exposure followed by its return to baseline at 60 min [29]. In our study we have also observed that the activation of pERK-1/2 by H_2_O_2_. These observations have been confirmed further by co-localization of p-ERK1/2 within the cytosol by immunoflorescence study.

Since ERK is known as a signal for mitogen-activated protein kinase (MAPK) pathway and has been shown to be involved in growth, differentiation and development in mammalian system [30], we sought to investigate its role following its induction by nicotine or hydrogen peroxide in AR42J cell proliferation employing MTT assay as reported earlier [10]. Our data show that while nicotine does promote significant growth within the first 48 hours of incubation in low serum media, H_2_O_2 _treated cells, on the other hand, do not show such an increase in proliferation and its effects on cell proliferation is similar to that of untreated cells (Figure 5). It has been suggested that the induction of p-ERK1/2 by H_2_O_2 _is a cell specific response [31], where H_2_O_2 _may be able to utilize multiple pathways to produce mitogenic effects depending on the cell type. Thus we surmise that even though the cells do not proliferate at a faster rate with H_2_O_2 _as observed for the cells exposed to nicotine, the rate of proliferation by H_2_O_2 _is similar to that of the control untreated cells. These complex regulatory mechanisms have been described previously by Watanabe et al [21], and others [32] Thus it is possible that induction of ERK signaling by H_2_O_2 _may be critical in the regulation of cellular protection in the early stage of cell response to oxidative stress. One other interpretation may be that H_2_O_2 _induced p-ERK activation and MDA formation are not involved in cellular proliferation.

Cell function studies have been conducted by stimulating the cells with a previously determined maximal dose of cholecystokinin (CCK). These experiments are aimed to determine whether there are any differences in cell function when they are exposed to nicotine or H_2_O_2 _at their optimum concentrations. While the CCK-induced amylase secretion with nicotine exposure is significantly higher than the unexposed cells, there is no increase in amylase secretion by H_2_O_2 _over and above that of control. It has been shown that CCK can evoke marked changes in pancreatic acinar cell mitochondrial activity and that CCK-8 evoked responses are blocked by H_2_O_2 _[33]. Impairment of mitochondrial activity in the presence of H_2_O_2 _(1 mM) may represent a mechanism by which cellular damage can occur leading to its dysfunction and pathology. The dose of 20 μM of H_2_O_2 _used in this study is nontoxic and therefore, the data from our studies showing normal cell function appear consistent with those observations.

H_2_O_2 _is a known oxidative agent and is used here as a biomarker by which its effect on AR42J cells can be directly compared to the effects of nicotine following its exposure. Functional and cell proliferation studies show a significant difference in the effects between nicotine and H_2_O_2 _on AR42J cells. This indicates that while nicotine exposure does result in the production of ROS within the cells, there are certain other key factors induced by nicotine that differentiates its effects from that of H_2_O_2_. In the current study, we have aimed to show that one of these key factors for cell injury is in the production or ROS. Since ROS has been shown to cause DNA single strand breakdown [34], it is reasonable to consider further investigation of the role of nicotine in signal transduction pathways and its oxidative role in pancreatic cell injury. In in-vivo studies, Wittel et al [35] investigated the effect of cigarette smoke inhalation in rats. Their studies showed that morphological damage to pancreas induced by inhalation of cigarette smoke may likely be mediated by alteration of acinar cell function. Our studies in in-vitro cell culture using nicotine as marker supports the observation made by Wittel et al [35].

## Authors' contributions

AW carried out all experiments, data analysis. and produced a draft of the manuscript. KBU participated in the design of the study and veified the statistical analysis. PC conceived and directed the study. All authors read and approved the final manuscript.
